# Forest Pruning Based on Branch Importance

**DOI:** 10.1155/2017/3162571

**Published:** 2017-06-01

**Authors:** Xiangkui Jiang, Chang-an Wu, Huaping Guo

**Affiliations:** ^1^School of Automation, Xi'an University of Posts and Telecommunication, Xi'an, Shaanxi 710121, China; ^2^School of Computer and Information Technology, Xinyang Normal University, Xinyang, Henan 464000, China

## Abstract

A forest is an ensemble with decision trees as members. This paper proposes a novel strategy to pruning forest to enhance ensemble generalization ability and reduce ensemble size. Unlike conventional ensemble pruning approaches, the proposed method tries to evaluate the importance of branches of trees with respect to the whole ensemble using a novel proposed metric called importance gain. The importance of a branch is designed by considering ensemble accuracy and the diversity of ensemble members, and thus the metric reasonably evaluates how much improvement of the ensemble accuracy can be achieved when a branch is pruned. Our experiments show that the proposed method can significantly reduce ensemble size and improve ensemble accuracy, no matter whether ensembles are constructed by a certain algorithm such as bagging or obtained by an ensemble selection algorithm, no matter whether each decision tree is pruned or unpruned.

## 1. Introduction

Ensemble learning is a very important research topic in machine learning and data mining. The basic heuristic is to create a set of learners and aggregate the prediction of each learner for classifying examples. Many approaches such as bagging [1], boosting [2], and COPEN [3] have been proposed to create ensembles, and the key to the success of these approaches is that base learners are accurate and diverse [4].

Ensemble methods have been applied to many applications such as image detection [5–7] and imbalanced learning problem [8]. However, an important drawback existing in ensemble learning approaches is that they try to train unnecessarily large ensembles. Large ensembles need a large memory for storing the bases learners and much response time for prediction. Besides, large ensemble may reduce its generalization ability instead of increasing the performance [9]. Therefore, a lot of researches to tackle this problem have been carried out, and the researches mainly focus on ensemble selection: selecting a subset of ensemble members for prediction, such as ordered-based ensemble selection methods [10–12] and greedy heuristic based ensemble selection methods [13–21]. The research results indicate that a well-designed ensemble selection method can reduce ensemble size and improve ensemble accuracy.

Besides ensemble selection, we can prune an ensemble through the following two approaches if ensemble members are decision trees: (1) pruning individual members separately and combining the pruned members together for prediction and (2) repeatedly pruning individual members by considering the overall performance of the ensemble. For the first strategy, many decision tree pruning methods such as those used in CART [22] and C4.5 [23] have been studied. Although pruning can simplify model structure, whether pruning can improve model accuracy is still a controversial topic in machine learning [24]. The second strategy coincides with the expectation of improving model generalization ability globally. However, this method has not been extensively studied. This paper focuses on this strategy and names the strategy as forest pruning (FP).

The major job of forest pruning is to define an effective metric evaluating the importance of a certain branch of trees. Traditional metrics can not be applied to forest pruning, since these metrics just consider the influence on a single decision tree when a branch is pruned. Therefore, we need a new metric for pruning forest. Our contributions in this paper are as follows:

- Introduce a new ensemble pruning strategy to prune decision tree based ensemble;
- propose a novel metric to measure the improvement of forest performance when a certain node grows into a subtree;
- present a new ensemble pruning algorithm with the proposed metric to prune a decision tree based ensemble. The ensemble can be learned by a certain algorithm or obtained by some ensemble selection method. Each decision tree can be pruned or unpruned.

Experimental results show that the proposed method can significantly reduce the ensemble size and improve its accuracy. This result indicates that the metric proposed in this paper reasonably measures the influence on ensemble accuracy when a certain node grows into a subtree.

The rest of this paper is structured as follows.  Section 2 provides a survey of ensemble of decision trees; Section 3 presents the formal description of forest trimming and the motivation of this study by an example.  Section 4 introduces a new forest pruning algorithm.  Section 5 reports and analyzes experimental results and we conclude the paper with simple remark and future work in Section 6.

## 2. Forests

A forest is an ensemble whose members are learned by decision tree learning method. Two approaches are often used to train a forest: traditional approaches and the methods specially designed for forests.

Bagging [1] and boosting [2] are the two most often used traditional methods to build forests. Bagging takes bootstrap samples of objects and trains a tree on each sample. The classifier votes are combined by majority voting. In some implementations, classifiers produce estimates of the posterior probabilities for the classes. These probabilities are averaged across the classifiers and the most probable class is assigned, called “average” or “mean” aggregation of the outputs. Bagging with average aggregation is implemented in Weka and used in the experiments in this paper. Since each individual classifier is trained on a bootstrap sample, the data distribution seen during training is similar to the original distribution. Thus, the individual classifiers in a bagging ensemble have relatively high classification accuracy. The factor encouraging diversity between these classifiers is the proportion of different examples in the training set. Boosting is a family of methods and Adaboost is the most prominent member. The idea is to boost the performance of a “weak” classifier (can be decision tree) by using it within an ensemble structure. The classifiers in the ensemble are added one at a time so that each subsequent classifier is trained on data which have been “hard” for the previous ensemble members. A set of weights is maintained across the objects in the data set so that objects that have been difficult to classify acquire more weight, forcing subsequent classifiers to focus on them.

Random forest [25] and rotation forest [26] are two important approaches specially designed for building forests. Random forest is a variant version of bagging. The forest is built again on bootstrap samples. The difference lies in the construction of the decision tree. The feature to split a node is selected as the best feature among a set of *M* randomly chosen features, where *M* is a parameter of the algorithm. This small alteration appeared to be a winning heuristic in that diversity was introduced without much compromising the accuracy of the individual classifiers. Rotation forest randomly splits the feature set into *K* subsets (*K* is a parameter of the algorithm) and Principal Component Analysis (PCA) [27] is applied to each subset. All principal components are retained in order to preserve the variability information in the data. Thus, *K* axis rotations take place to form the new features and rotation forest building a tree using all training set in the new space defined by a given new feature space.

## 3. Problem Description and Motivation

### 3.1. Problem Description

Let *D* = {(*x*_*i*_, *y*_*i*_)∣*i* = 1,2,…, *N*} be a data set, and let *F* = {*T*_1_,…, *T*_*M*_} be an ensemble with decision tree, *T*_*i*_, learning from *D*. Denote by *v* ∈ *T*_*t*_ a node in tree *T* and by *E*(*v*) ∈ *D*, the set of the examples reaching *v* from the root of *T*, root(*T*). Suppose each node *v* ∈ *T* contains a vector (*p*_1_^*v*^, *p*_2_^*v*^,…, *p*_*K*_^*v*^), where *p*_*k*_^*v*^ is the proportion of the examples in *E*(*v*) associated with label *k*. If *v* ∈ *T*_*i*_ is a leaf and **x**_*i*_ ∈ *E*(*v*), the prediction of *T*_*j*_ on **x**_*i*_ is

(1) $$\begin{array}{c} T_{j}\left(\;x_{i}\;\right)=\arg\underset{k}{\max}\;p_{k}^{v}. \end{array}$$

Similarly, for each example **x**_*j*_ to be classified, ensemble *F* returns a vector (*p*_*j*1_, *p*_*j*2_,…, *p*_*jK*_) indicating that **x**_*j*_ belongs to label *k* with probability *p*_*jk*_, where

(2) $$\begin{array}{c} p_{jk}=\frac{1}{M}\overset{M}{\underset{j=1}{\sum }}p_{jk}^{\left(i\right)},\;k=1,2,\ldots ,K. \end{array}$$

The prediction of *F* on **x**_*j*_ is *F*(**x**_*j*_) =  argmax_*k*_⁡*p*_*jk*_.

Now, our problem is, given a forest *F* with *M* decision trees, how to prune each tree to reduce *F*'s size and improve its accuracy, where *F* is either constructed by some algorithm or obtained by some ensemble selection method.

### 3.2. Motivation

First, let us look at an example, which shows the possibility that forest trimming can improve ensemble accuracy.

**Example 1 .**

**Figure 1:**
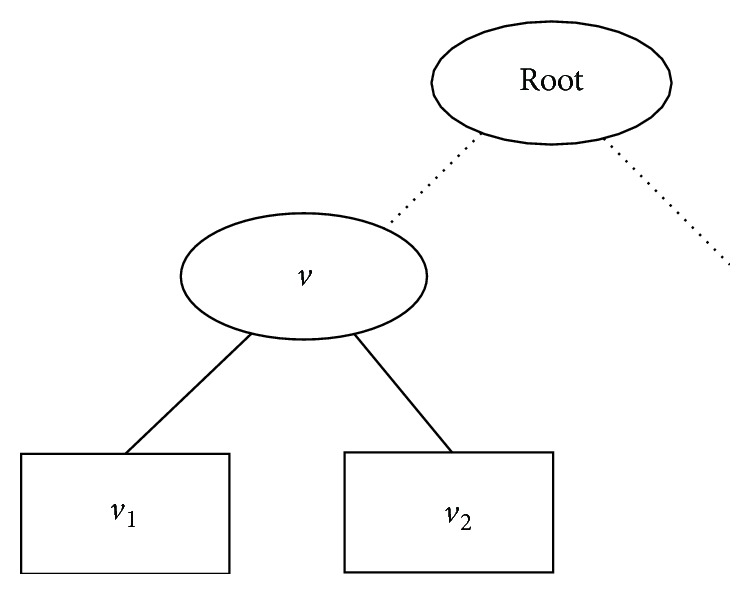
Decision tree *T*_0_. *v* is a test node and *v*_1_ and *v*_2_ are two leaves.

Let *F* = {*T*_0_, *T*_1_,…, *T*_9_} be a forest with ten decision trees, where *T*_1_ is shown in Figure 1. Suppose that *p*_1_^*v*^ = 0.60, *p*_2_^*v*^ = 0.40; *p*_1_^*v*_1_^ = 1.00, *p*_2_^*v*_1_^ = 0.00; *p*_1_^*v*_2_^ = 0.20 and *p*_2_^*v*_2_^ = 0.80. Let ten examples **x**_0_, **x**_1_,…, **x**_9_ reach node *v*, where **x**_0_,…, **x**_5_ associate with label 1 and **x**_6_,…, **x**_9_ associate with label 2. Assume examples **x**_0_, **x**_1_,…, **x**_4_ reach leaf node *v*_1_, and **x**_5_,…, **x**_9_ reach leaf node *v*_1_.

Obviously, for *T*_0_, we can not prune the children of node *v*, since treating *v* as a leaf would lead to more examples incorrectly classified by *T*_0_.

Assume that *F*'s predictions on **x**_0_, **x**_1_,…, **x**_9_ are as follows:

(3) p01=0.65,p11=0.70,p21=0.70,p31=0.65,p41=0.80,p51=0.49,p61=0.30,p71=0.19,p81=0.20,p91=0.30,p02=0.35,p12=0.30,p22=0.30,p32=0.35,p42=0.20,p52=0.51,p62=0.70,p72=0.81,p82=0.80,p92=0.70.

where *p*_*jk*_ is the probability of **x**_*j*_ associated with label *k*. From *F*'s predictions shown above, we have that **x**_6_ is incorrectly classified by *F*. Update *T*_0_ to *T*_0_′ by pruning *v*'s children and update *F* to *F*′ = {*T*_0_′, *T*_1_,…, *T*_9_}. A simple calculation tells us that, for the ten examples, *F*′ returns:

(4) p01=0.61,p11=0.65,p21=0.65,p31=0.65,p41=0.75,p51=0.52,p61=0.33,p71=0.22,p81=0.23,p91=0.33,p02=0.40,p12=0.35,p22=0.35,p32=0.35,p42=0.25,p52=0.48,p62=0.67,p72=0.78,p82=0.77,p92=0.67.

It is easy to see that *F*′ correctly classifies all of the ten examples.

This example shows that if a single decision tree is considered, maybe it should not be pruned any more. However, for the forest as a whole, it is still possible to prune some branches of the decision tree, and this pruning will probably improve the ensemble accuracy instead of reducing it.

Although the example above is constructed by us, similar cases can be seen everywhere when we study ensembles further. It is this observation that motivates us to study forest trimming methods. However, more efforts are needed to turn possibility into feasibility. Further discussions about this problem will be presented in the next section.

## 4. Forest Pruning Based on Branch Importance

### 4.1. The Proposed Metric and Algorithm Idea

To avoid trapping in detail too early, we assume that *I*(*v*, *F*, **x**_*j*_) has been defined, which is the importance of node *v* when forest *F* classifies example **x**_*j*_. If **x**_*j*_ ∉ *E*(*v*), then *I*(*v*, *F*, **x**_*j*_) = 0. Otherwise, the details of the definition of *I*(*v*, *F*, **x**_*j*_) are presented in Section 3.2.

Let *T* ∈ *F* be a tree and let *v* ∈ *T* be a node. The importance of *v* with respect to forest *F* is defined as

(5) $$\begin{array}{c} I\left(\;v,F\;\right)=\underset{x_{j}\in D^{\prime }}{\sum }I\left(\;v,F,x_{j}\;\right)=\underset{x_{j}\in E\left(v\right)}{\sum }I\left(\;v,F,x_{j}\;\right), \end{array}$$

where *D*′ is a pruning set and *E*(*v*) is the set of the example in *D*′ reaching node *v* from root(*T*). *I*(*v*, *F*) reflects the impact of node *v* on *F'*s accuracy.

Let *L*(*v*) be the set of leaf nodes of branch(*v*), the branch (subtree) with *v* as the root. The contribution of branch(*v*) to *F* is defined as

(6) $$\begin{array}{c} I\left(\;branch\left(\;v\;\right),F\;\right)=\underset{v^{\prime }\in L\left(v\right)}{\sum }I\left(\;v^{\prime },F\;\right), \end{array}$$

which is the sum of the importance of leaves in branch(*v*).

Let *v* ∈ *T* be a nonterminal node. The importance gain of *v* to *F* is defined by the importance difference between branch(*v*) and node *v*, that is,

(7) $$\begin{array}{c} IG\left(\;v,F\;\right)=I\left(\;branch\left(\;v\;\right)\;\right)-I\left(\;v,F\;\right), \end{array}$$

IG(*v*, *F*) can be considered as the importance gain of branch(*v*), and its value reflects how much improvement of the ensemble accuracy is achieved when *v* grows into a subtree. If IG(*v*, *F*) > 0, then this expansion is helpful to improve *F'*s accuracy. Otherwise it is unhelpful to improve or even reduce *F'*s accuracy.

The idea of the proposed method of pruning ensemble of decision trees is as follows. For each nonterminal node *v* in each tree *T*, calculate its importance gain IG(*v*, *F*) on the pruning set. If IG(*v*, *F*) is smaller than a threshold, prune branch(*v*) and treat *v* as a leaf. This procedure continues until all decision trees can not be pruned.

Before presenting the specific details of the proposed algorithm, we introduce how to calculate *I*(*v*, *F*, **x**_*j*_) in the next subsection.

### 4.2. Con(*v*, *F*, **x**_*j*_) Calculation

Let *h* be a classifier and let *S* be an ensemble. Partalas et al. [28, 29] identified that the prediction of *h* and *S* on an example **x**_*j*_ can be categorized into four cases: (1)  *e*_*tf*_: *h*(**x**_*i*_) = *y*_*i*_∧*S*(**x**_*i*_) ≠ *y*_*i*_, (2)  *e*_*tt*_: *h*(**x**_*i*_) = *y*_*i*_∧*S*(**x**_*i*_) = *y*_*i*_, (3)  *e*_*ft*_: *h*(**x**_*i*_) ≠ *y*_*i*_∧*S*(**x**_*i*_) = *y*_*i*_, (4)  *e*_*ft*_: *h*(**x**_*i*_) ≠ *y*_*i*_∧*S*(**x**_*i*_) ≠ *y*_*i*_. They concluded that considering all four cases is crucial to design ensemble diversity metrics.

Based on the four cases above, Lu et al. [11] introduced a metric, IC_*i*_^(*j*)^, to evaluate the contribution of the *i*th classifier to *S* when *S* classifies the *j*th instance. Partalas et al. [28, 29] introduced a measure called Uncertainty Weighted Accuracy, UWA_*D*_(*h*, *S*, **x**_*j*_), to evaluate *h'*s contribution when *S* classifies example **x**_*j*_.

Similar to the discussion above, we define

(8) etfv=xj ∣ xj∈Ev∧Txj=yj∧Fxj≠yj,ettv=xj ∣ xj∈Ev∧Tixj=yj∧Fxj=yj,eftv=xj ∣ xj∈Ev∧Tixj≠yj∧Fxj=yj,effv=xj ∣ xj∈Ev∧Tixj≠yj∧Fxj≠yj.

In the following discussions, we assume that *v* ∈ *T* and **x**_*j*_ ∈ *E*(*v*). Let *f*_*m*_ and *f*_*s*_ be the subscripts of the largest element and the second largest element in {*p*_*j*1_,…, *p*_*jK*_}, respectively. Obviously, *f*_*m*_ is the label of **x**_*j*_ predicted by ensemble *F*. Similarly, let *t*_*m*_ = arg⁡max_*k*_(*p*_1_^*v*^,…, *p*_*K*_^*v*^). If *v* is a leaf node, then *t*_*m*_ is the label of **x**_*j*_ predicted by decision tree *T*. Otherwise, *t*_*m*_ is the label of **x**_*j*_ predicted by *T*′, where *T*′ is the decision tree obtained from *T* by pruning branch(*v*). For simplicity, we call *t*_*m*_ the label of **x**_*j*_ predicted by node *v* and say node *v* correctly classifies **x**_*j*_ if *t*_*m*_ = *y*_*j*_.

We define *I*(*v*, *F*, **x**_*j*_) based on the four cases in formula (8), respectively. If **x**_*j*_ ∈ *e*_*tf*_(*v*) or **x**_*j*_ ∈ *e*_*tt*_(*v*), then Con(*v*, *F*, **x**_*j*_) ≥ 0, since *v* correctly classifies **x**_*j*_. Otherwise, Con(*v*, *F*, **x**_*j*_) < 0, since *v* incorrectly classifies **x**_*j*_.

For **x**_*j*_ ∈ *e*_*tf*_(*v*), Con(*v*, *F*, **x**_*j*_) is defined as

(9) $$\begin{array}{c} I\left(\;v,F,x_{j}\;\right)=\frac{p_{t_{m}}^{v}-p_{f_{m}}^{v}}{M\left(p_{jf_{m}}-p_{jt_{m}}+1/M\right)}, \end{array}$$

where *M* is the number of base classifiers in *F*. Here, *t*_*m*_ = *y*_*j*_ and *f*_*m*_ ≠ *y*_*j*_, then *p*_*t*_*m*__^*v*^ ≥ *p*_*f*_*m*__^*v*^, *p*_*jf*_*m*__ ≥ *p*_*jt*_*m*__, and thus 0 ≤ Con(*v*, *F*, **x**_*j*_) ≤ 1. Since *p*_*f*_*m*__^*v*^ is the contribution of node *v* to the probability that *F* correctly predicates **x**_*j*_ belonging to class *t*_*m*_ while *p*_*f*_*m*__^*v*^ is the contribution of node *v* to *p*_*jf*_*m*__, the probability that *F* incorrectly predicates **x**_*j*_ belongs to class *f*_*m*_, (*p*_*t*_*m*__^*v*^ − *p*_*f*_*m*__^*v*^)/*M* can be considered as the net importance of node *v* when *F* classifies **x**_*j*_. *p*_*jf*_*m*__ − *p*_*jt*_*m*__ is the weight of *v'*s net contribution, which reflects the importance of node *v* for classifying **x**_*j*_ correctly. The constant 1/*M* is to avoid *p*_*jf*_*m*__ − *p*_*jt*_*m*__ being zero or too small.

For **x**_*j*_ ∈ *e*_*tf*_(*v*), Con(*v*, *F*, **x**_*j*_) is defined as

(10) $$\begin{array}{c} I\left(\;v,F,x_{j}\;\right)=\frac{p_{t_{m}}^{v}-p_{t_{s}}^{v}}{M\left(p_{jf_{m}}-p_{jf_{s}}+1/M\right)}. \end{array}$$

Here, 0 ≤ Con(*v*, *F*, **x**_*j*_) ≤ 1. In this case, both *v* and *F* correctly classify **x**_*j*_. We treat (*p*_*t*_*m*__^*v*^ − *p*_*t*_*s*__^*v*^)/*M* as the net contribution of node *v* to *F* and **x**_*j*_, and *p*_*jf*_*m*__ − *p*_*jf*_*s*__ as the weight of *v'*s net contribution.

For **x**_*j*_ ∈ *e*_*ft*_(*v*), Con(*v*, *F*, **x**_*j*_) is defined as

(11) $$\begin{array}{c} I\left(\;v,F,x_{j}\;\right)=-\frac{p_{t_{m}}^{v}-p_{f_{m}}^{v}}{M\left(p_{jf_{m}}-p_{jf_{s}}+1/M\right)}. \end{array}$$

It is easy to prove −1 ≤ Con(*v*, *F*, **x**_*j*_) ≤ 0. This case is opposed to the first case. In this case, we treat −(*p*_*t*_*m*__^*v*^ − *p*_*f*_*m*__^*v*^)/*M* as the net contribution of node *v* to *F* and **x**_*j*_, and *p*_*jf*_*m*__ − *p*_*jf*_*s*__ as the weight of *v'*s net contribution.

For **x**_*j*_ ∈ *e*_*ff*_(*v*), Con(*v*, *F*, **x**_*j*_) is defined as

(12) $$\begin{array}{c} I\left(\;v,F,x_{j}\;\right)=-\frac{p_{t_{m}}^{v}-p_{y_{j}}^{v}}{M\left(p_{jf_{m}}-p_{jy_{j}}+1/M\right)}, \end{array}$$

where *y*_*j*_ ∈ {1,…, *K*} is the label of **x**_*j*_, −1 ≤ Con(*v*, *F*, **x**_*j*_) ≤ 0. In this case, both *v* and *F* incorrectly classify **x**_*j*_, namely, *t*_*m*_ ≠ *y*_*j*_ and *f*_*m*_ ≠ *y*_*j*_. We treat −(*p*_*t*_*m*__^*v*^ − *p*_*y*_*j*__^*v*^)/*M* as the net contribution of node *v* to *F* and **x**_*j*_, and *p*_*jf*_*m*__ − *p*_*jy*_*j*__ as the weight of *v'*s net contribution.

### 4.3. Algorithm

The specific details of forest pruning (FP) are shown in Algorithm 1, where

 
*D*′ is a pruning set containing *n* instances,
 
*p*_*jk*_ is the probability that ensemble *F* predicts **x**_*j*_ ∈ *D*′ associated with label *k*,
 
*p*_*ijk*_ is the probability that current tree *T*_*i*_ predicts **x**_*j*_ ∈ *D*′ associated with label *k*,
 
*I*_*v*_ is a variant associated with node *v* to save *v*'s importance,
 
*I*_br(*v*)_ is a variant associated with node *v* to save the contribution of branch(*v*).

FP first calculates the probability of *F*'s prediction on each instance **x**_*j*_ (lines (1)~(2)). Then it iteratively deals with each decision tree *T*_*i*_ (lines (3)~(14)). Lines (4)~(10) calculate the importance of each node *v* ∈ *T*_*i*_, where *I*(*v*, *F*, **x**_*j*_) in line (10) is calculated using one of the equations (9)~(12) based on the four cases in equation (8). Line (11) calls PruningTree(*v*) to recursively prune *T*_*i*_. Since forest *F* has been changed after pruning *T*_*i*_, we adjust *F'*s prediction in lines (12)–(14). Lines (3)–(14) can be repeated many times until all decision trees can not be pruned. Experimental results show that forest performance is stable after this iteration is executed 2 times.

The recursive procedure PruningTree(*v*) adopts a bottom-up fashion to prune the decision tree with *v* as the root. After pruning  branch(*v*)  (subtree(*v*)), *I*_*v*_ saves the sum of the importance of leaf nodes in branch(*v*). Then *I*(branch(*v*), *F*) is equal to the sum of importance of the tree with *v* as root. The essence of using *T*_*i*_'s root to call PruningTree is to travel *T*_*i*_. If current node *v* is a nonleaf, the procedure calculates *v*'s importance gain IG, saves into *I*_*v*_ the importance sum of the leaves of branch(*v*) (lines (2)~(7)), and determines pruning branch(*v*) or not based on the difference between CG and the threshold value *δ* (lines (8)~(9)).

### 4.4. Discussion

Suppose pruning set *D*′ contains *n* instances, forest *F* contains *M* decision trees, and *d*_max_ is the depth of the deepest decision tree in *F*. Let |*T*_*i*_| be the number of nodes in decision tree *T*_*i*_, and *t*_max_ = max_1≤*i*≤*M*_⁡(|*T*_*i*_|). The running time of FP is dominated by the loop from lines (4) to (19). The loop from lines (5) to (7) traverses *T*_*i*_, which is can be done in *O*(*t*_max_); the loop from lines (8) to (14) searches a path of *T*_*i*_ for each instance in *D*′, which is complexity of *O*(*nd*_max_); the main operation of PruningTree(root(*T*_*i*_)) is a complete traversal of *T*_*i*_, whose running time is *O*(*t*_max_); the loop from lines (16) to (18) scans a linear list of length *n* in *O*(*n*). Since *t*_max_ ≪ *nd*_max_, we conclude the running time of FP is *O*(*nMd*_max_). Therefore, FP is a very efficient forest pruning algorithm.

Unlike traditional metrics such as those used by CART [22] and C4.5 [23], the proposed measure uses a global evaluation. Indeed, this measure involves the prediction values that result from a majority voting of the whole ensemble. Thus, the proposed measure is based on not only individual prediction properties of ensemble members but also the complementarity of classifiers.

From equations (9), (10), (11), and (12), our proposed measure takes into account both the correctness of predictions of current classifier and the predictions of ensemble and the measure deliberately favors classifiers with a better performance in classifying the samples on which the ensemble does not work well. Besides, the measure considers not only the correctness of classifiers, but also the diversity of ensemble members. Therefore, using the proposed measure to prune an ensemble leads to significantly better accuracy results.

## 5. Experiments

### 5.1. Experimental Setup

19 data sets of which the details are shown in Table 1 are randomly selected from UCI repertory [30], where #Size, #Attrs, and #Cls are the size, attribute number, and class number of each data set, respectively. We design four experiments to study the performance of the proposed method (forest pruning, FP):

- The first experiment studies FP's performance versus the times of running FP. Here, four data sets, that is, autos, balance-scale, German-credit, and pima, are selected as the representatives, and each data set is randomly divided into three subsets with equal size, where one is used as the training set, one as the pruning set, and the other one as the testing set. We repeat 50 independent trials on each data set. Therefore a total of 300 trials of experiments are conducted.
- The second experiment is to evaluate FP's performance versus FL's size (number of base classifiers). The experimental setup of data sets is the same as the first experiment.
- The third experiment aims to evaluate FP's performance on pruning ensemble constructed by bagging [1] and random forest [26]. Here, tenfold cross-validation is employed: each data set is divided into tenfold [31, 32]. For each one, the other ninefold is to train model, and the current one is to test the trained model. We repeat 10 times the tenfold cross-validation and thus, 100 models are constructed on each data set. Here, we set the training set as the pruning set. Besides, algorithm rank is used to further test the performance of algorithms [31–33]: on a data set, the best performing algorithm gets the rank of 1.0, the second best performing algorithm gets the rank of 2.0, and so on. In case of ties, average ranks are assigned.
- The last experiment is to evaluate FP's performance on pruning the subensemble obtained by ensemble selection method. EPIC [11] is selected as the candidate of ensemble selection methods. The original ensemble is a library with 200 base classifiers, and the size of subsembles is 30. The setup of data sets is the same as the third experiment.

In the experiments, bagging is used to train original ensemble, and the base classifier is J48, which is a Java implementation of C4.5 [23] from Weka [34]. In the third experiment, random forest is also used to build forest. In the last three experiments, we run FP two times.

### 5.2. Experimental Results

**Figure 2:**
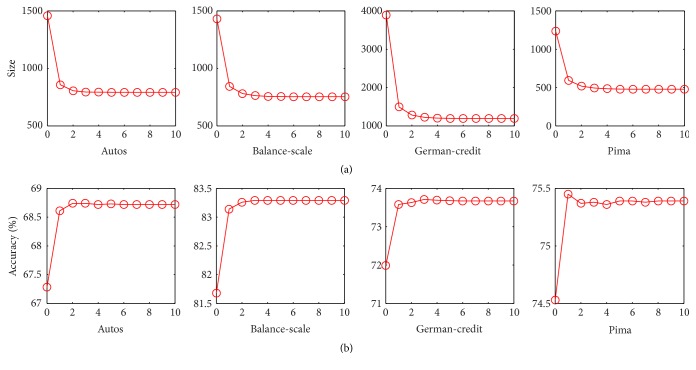
Results on data sets. (a) Forest size (node number) versus the times of running FP. (b) Forest accuracy versus the times of running FP.

The first experiment is to investigate the relationship of the performance of the proposed method (FP) and the times of running FP. In each trial, we first use bagging to learn 30 unpruned decision trees as a forest and then iteratively run lines (3)~(14) of FP many times to trim the forest. More experimental setup refers to Section 5.1. The corresponding results are shown in Figure 2, where the top four subfigures are the variation trend of forest nodes number with the iteration number increasing, and the bottom four are the variation trend of ensemble accuracy. Figure 2 shows that FP significantly reduces forests size (almost 40%~60% of original ensemble) and significantly improves their accuracy. However, the performance of FP is almost stable after two iterations. Therefore, we set iteration number to be 2 in the following experiments.

**Figure 3:**
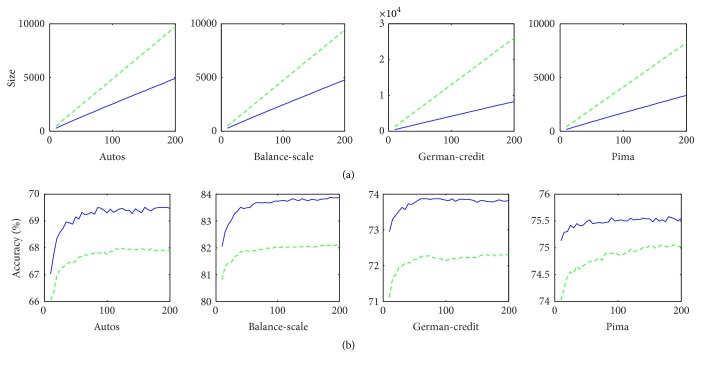
Results on data sets. (a) Forest size (node number) versus the number of decision trees. (b) Forest accuracy versus the number of decision trees. Solid curves and dash curves represent the performance of FP and bagging, respectively.

The second experiment aims at investigating the performance of FP on pruning forests with different scales. The number of decision trees grows gradually from 10 to 200. More experimental setup refers to Section 5.1. The experimental results are shown in Figure 3, where the top four subfigures are the comparison between pruned and unpruned ensembles with the growth of the number of decision trees, and the bottom four are the comparison of ensemble accuracy. As shown in Figure 3, for each data set, the rate of forest nodes pruned by FP keeps stable and forests accuracy improved by FP is also basically unchanged, no matter how many decision trees are constructed.

The third experiment is to evaluate the performance of FP on pruning the ensemble constructed by ensemble learning method. The setup details are shown in Section 5.1. Tables 2, 3, 4, and 5 show the experimental results of compared methods, respectively, where Table 2 reports the mean accuracy and the ranks of algorithms, Table 3 reports the average ranks using nonparameter Friedman test [32] (using STAC Web Platform [33]), Table 4 reports the comparing results using post hoc with Bonferroni-Dunn (using STAC Web Platform [33]) of 0.05 significance level, and Table 5 reports the mean node number and standard deviations. Standard deviations are not provided in Table 2 for clarity. The column of “FP” of Table 2 is the results of pruned forest and, “bagging” and “random forest” are the results of unpruned forests constructed by bagging and random forest, respectively. In Tables 3 and 4, Alg1, Alg2, Alg3, Alg4, Alg5, and Alg6 indicate PF pruning bagging with unpruned C4.5, bagging with unpruned C4.5, PF pruning bagging with pruned C4.5, bagging with pruned C4.5, PF pruning random forest, and random forest. From Table 2, FP significantly improves ensemble accuracy in most of the 19 data sets, no matter whether the individual classifiers are pruned or unpruned, no matter whether the ensemble is constructed by bagging or random forest. Besides, Table 2 shows that the ranks of FP always take place of best three methods in these data sets. Tables 3 and 4 validate the results in Table 2, where Table 3 shows that the average rank of PF is much small than other methods and Table 4 shows that, compared with other methods, PF shows significant better performance. Table 5 shows FP is significantly smaller than bagging and random forest, no matter whether the individual classifier is pruned or not.

The last experiment is to evaluate the performance of FP on pruning subensembles selected by ensemble selection method EPIC.  Table 6 shows the results on the 19 data sets, where left and right are the accuracy and size, respectively. As shown in Table 6, FP can further significantly improve the accuracy of subensembles selected by EPIC and reduce the size of the subensembles.

## 6. Conclusion

An ensemble with decision trees is also called forest. This paper proposes a novel ensemble pruning method called forest pruning (FP). FP prunes trees' branches based on the proposed metric called branch importance, which indicates the importance of a branch (or a node) with respect to the whole ensemble. In this way, FP achieves reducing ensemble size and improving the ensemble accuracy.

The experimental results on 19 data sets show that FP significantly reduces forest size and improves its accuracy in most of the data sets, no matter whether the forests are the ensembles constructed by some algorithm or the subensembles selected by some ensemble selection method, no matter whether each forest member is a pruned decision tree or an unpruned one.

## Figures and Tables

**Algorithm 1:**
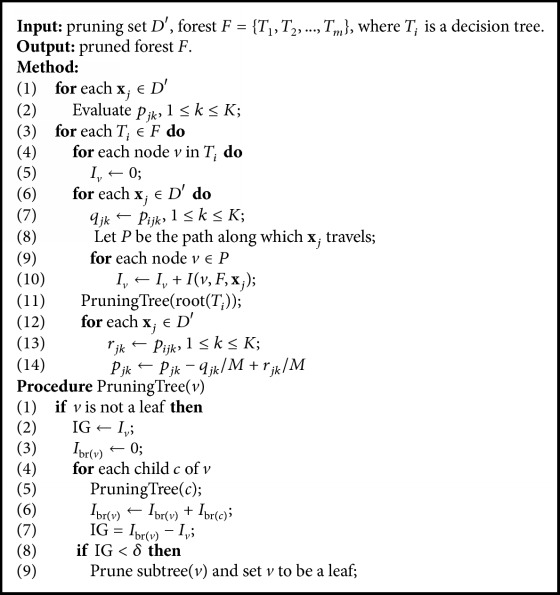
The procedure of forest pruning.

**Table 1:** The details of data sets used in this paper.

Data set	#Attrs	#Size	#Cls
Australian	226	70	24
Autos	205	26	6
Backache	180	33	2
Balance-scale	625	5	3
Breast-cancer	268	10	2
Cars	1728	7	4
Credit-rating	690	16	2
German-credit	1000	21	2
Ecoli	336	8	8
Hayes-roth	160	5	4
Heart-c	303	14	5
Horse-colic	368	24	2
Ionosphere	351	35	2
Iris	150	5	3
Lymph	148	19	4
Page-blocks	5473	11	5
Pima	768	9	2
prnn-fglass	214	10	6
Vote	439	17	2

**Table 2:** The accuracy of FP, bagging, and random forest. ∙ represents that FP outperforms bagging in pairwise *t*-tests at 95% significance level and denotes that FP is outperformed by bagging.

Dataset	Unpruned C4.5		Pruned C4.5		PF	RF
	PF	Bagging	PF	Bagging		
Australian	87.14 (2.0)	86.09 (5.0)^∙^	86.80 (3.0)	85.86 (6.0)^∙^	87.21 (1.0)	86.14 (4.0)^∙^
Autos	74.40 (2.0)	73.30 (4.0)^∙^	74.20 (3.0)	73.20 (5.0)^∙^	74.72 (1.0)	73.10 (6.0)^∙^
Backache	85.07 (3.0)	83.17 (5.5)^∙^	85.89 (1.0)	83.17 (5.5)^∙^	85.21 (2.0)	83.22 (4.0)^∙^
Balance-scale	78.89 (3.0)	75.07 (6.0)^∙^	79.79 (1.0)	76.64 (4.0)^∙^	79.65 (2.0)	76.32 (5.0)^∙^
Breast-cancer	69.98 (2.0)	67.10 (5.0)^∙^	69.97 (3.0)	66.58 (6.0)^∙^	70.11 (1.0)	68.88 (4.0)^∙^
Cars	86.51 (4.0)	86.78 (2.0)	86.88 (1.0)	86.28 (5.0)	86.55 (3.0)	86.11 (6.0)
Credit-rating	86.44 (2.0)	85.54 (4.0)^∙^	86.34 (3.0)	85.43 (5.0)^∙^	86.82 (1.0)	85.42 (6.0)^∙^
German-credit	75.33 (1.0)	73.83 (4.0)^∙^	74.86 (3.0)	73.11 (6.0)^∙^	75.22 (2.0)	73.18 (5.0)^∙^
Ecoli	84.47 (2.0)	83.32 (6.0)^∙^	84.20 (3.0)	83.40 (5.0)^∙^	84.52 (1.0)	83.89 (4.0)^∙^
Hayes-roth	78.75 (3.0)	78.63 (5.0)	78.77 (1.0)	76.31 (6.0)^∙^	78.76 (2.0)	77.77 (4.0)
Heart-c	80.94 (2.0)	80.34 (5.0)	81.01 (1.0)	80.27 (6.0)	80.90 (3.0)	80.87 (4.0)
Horse-colic	84.52 (1.0)	83.29 (6.0)^∙^	84.33 (2.0)	83.42 (5.0)^∙^	84.31 (3.0)	83.99 (4.0)
Ionosphere	93.99 (1.0)	93.93 (2.0)	93.59 (6.0)	93.71 (4.0)	93.87 (3.0)	93.56 (5.0)
Iris	93.55 (6.0)	94.24 (4.0)	94.52 (3.0)	94.53 (2.0)	94.21 (5.0)	94.62 (1.0)
Lymphography	83.81 (5.0)	83.43 (6.0)	84.55 (2.0)	84.53 (3.0)	84.38 (4.0)	84.82 (1.0)
Page-blocks	97.03 (4.5)	97.04 (2.5)	97.04 (2.5)	97.06 (1.0)	97.03 (4.5)	97.01 (6.0)
Pima	75.09 (3.0)	74.27 (4.0)^∙^	75.46 (1.0)	74.06 (5.0)^∙^	75.43 (2.0)	73.21 (6.0)^∙^
prnn-fglass	78.14 (4.0)	78.46 (1.0)	77.62 (6.0)	77.84 (5.0)	78.18 (3.0)	78.32 (2.0)
Vote	95.77 (1.0)	95.13 (6.0)^∙^	95.67 (3.0)	95.33 (4.0)	95.72 (2.0)	95.31 (5.0)

**Table 3:** The ranks of algorithms using Friedman test, where Alg1, Alg2, Alg3, Alg4, Alg5, and Alg6 indicate PF pruning bagging with unpruned C4.5, bagging with unpruned C4.5, PF pruning bagging with pruned C4.5, bagging with pruned C4.5, PF pruning random forest, and random forest.

Algorithm	Alg5	Alg3	Alg1	Alg2	Alg6	Alg4
Ranks	2.39	2.50	2.71	4.32	4.42	4.66

**Table 4:** The testing results using post hoc, Alg1, Alg2, Alg3, Alg4, Alg5, and Alg6 indicate PF pruning bagging with unpruned C4.5, bagging with unpruned C4.5, PF pruning bagging with pruned C4.5, bagging with pruned C4.5, PF pruning random forest, and random forest.

Comparison	Statistic	*p* value
Alg1 versus Alg2	2.64469	0.04088
Alg3 versus Alg4	3.55515	0.00189
Alg5 versus Alg6	3.33837	0.01264

**Table 5:** The size (node number) of PF and bagging. ∙ denotes that the size of PF is significantly smaller than the corresponding comparing method.

Dataset	Unpruned C4.5		Pruned C4.5		PF-RF	RF
	PF	Bagging	PF	Bagging		
Australian	4440.82 ± 223.24	5950.06 ± 210.53^∙^	2194.71 ± 99.65	2897.88 ± 98.66^∙^	1989.67 ± 99.65	2653.88 ± 99.61^∙^
Autos	1134.83 ± 193.45	1813.19 ± 183.49^∙^	987.82 ± 198.22	1523.32 ± 193.22^∙^	954.26 ± 198.22	1429.12 ± 182.21^∙^
Backache	1162.79 ± 96.58	1592.80 ± 75.97^∙^	518.77 ± 40.49	764.24 ± 37.78^∙^	522.74 ± 40.49	789.23 ± 45.62^∙^
Balance-scale	3458.52 ± 74.55	4620.58 ± 78.20^∙^	3000.44 ± 71.76	3762.60 ± 65.55^∙^	2967.44 ± 71.76	3763.19 ± 79.46^∙^
Breast-cancer	2164.64 ± 156.41	3194.20 ± 144.95^∙^	843.96 ± 129.44	1189.33 ± 154.08^∙^	886.66 ± 129.44	1011.21 ± 148.92^∙^
Cars	1741.68 ± 60.59	2092.20 ± 144.95^∙^	1569.11 ± 57.55	1834.91 ± 46.80^∙^	1421.32 ± 56.65	1899.92 ± 68.88^∙^
Credit-rating	4370.65 ± 219.27	5940.51 ± 223.51^∙^	2168.11 ± 121.51	2904.40 ± 99.73^∙^	2015.21 ± 140.58	2650.40 ± 102.13^∙^
German-credit	9270.75 ± 197.62	11464.19 ± 168.63^∙^	4410.11 ± 114.94	5421.60 ± 107.24^∙^	4311.54 ± 124.68	5340.60 ± 217.48^∙^
Ecoli	1366.62 ± 61.68	1736.52 ± 64.91^∙^	1304.30 ± 54.39	1611.02 ± 56.31^∙^	1324.30 ± 54.42	1820.02 ± 88.74^∙^
Hayes-roth	498.65 ± 28.99	697.58 ± 40.87^∙^	272.30 ± 45.11	308.48 ± 53.86^∙^	264.24 ± 46.46	299.48 ± 63.84^∙^
Heart-c	1503.46 ± 65.47	1946.94 ± 62.52^∙^	647.89 ± 102.15	974.93 ± 129.83^∙^	647.89 ± 102.15	1032.93 ± 111.57^∙^
Horse-colic	2307.67 ± 106.99	3625.23 ± 116.63^∙^	684.29 ± 106.35	974.93 ± 129.83^∙^	647.89 ± 102.15	743.25 ± 120.43^∙^
Ionosphere	552.49 ± 61.41	680.43 ± 69.95^∙^	521.83 ± 58.01	634.73 ± 64.44^∙^	542.58 ± 96.02	665.84 ± 66.44^∙^
Iris	168.46 ± 111.12	222.66 ± 150.42^∙^	144.52 ± 97.26	191.84 ± 133.12^∙^	133.24 ± 98.32	212.55 ± 129.47^∙^
Lymphography	1089.87 ± 67.16	1394.37 ± 61.85^∙^	711.62 ± 37.61	856.44 ± 30.83^∙^	724.53 ± 37.61	924.33 ± 50.78^∙^
Page-blocks	1420.05 ± 278.51	2187.45 ± 555.02^∙^	1394.11 ± 600.06	2092.93 ± 403.79^∙^	1401.11 ± 588.03	2134.40 ± 534.97^∙^
Pima	2202.41 ± 674.18	2776.77 ± 852.95^∙^	2021.19 ± 698.02	2481.64 ± 747.19^∙^	1927.67 ± 625.27	2521.43 ± 699.82^∙^
prnn-fglass	1219.98 ± 39.85	1398.62 ± 36.29^∙^	1145.20 ± 39.76	1269.28 ± 35.52^∙^	1098.18 ± 34.26	1314.05 ± 60.97^∙^
Vote	303.06 ± 124.00	527.80 ± 225.05^∙^	174.04 ± 77.61	276.00 ± 127.46^∙^	182.14 ± 76.21	288.33 ± 113.76^∙^

**Table 6:** The performance of FP on pruning subensemble obtained by FP on bagging. ∙ represents that FP is significantly better (or smaller) than EPIC in pairwise *t*-tests at 95% significance level and denotes that FP is significantly worse (or larger) than EPIC.

Dataset	Error rate		Size	
	PF	EPIC	PF	EIPC
Australian	86.83 ± 3.72	86.22 ± 3.69^∙^	2447.50 ± 123.93	3246.16 ± 116.07^∙^
Autos	84.83 ± 4.46	82.11 ± 5.89^∙^	708.01 ± 54.55	931.44 ± 51.16^∙^
Backache	84.83 ± 4.46	82.11 ± 5.89^∙^	708.01 ± 54.55	931.44 ± 51.16^∙^
Balance-scale	79.74 ± 3.69	78.57 ± 3.82^∙^	3277.76 ± 85.07	4030.82 ± 94.67^∙^
Breast-cancer	70.26 ± 7.24	67.16 ± 8.36^∙^	843.96 ± 129.44	1189.33 ± 154.08^∙^
Cars	87.02 ± 5.06	86.83 ± 5.04	178.32 ± 60.44	2022.81 ± 53.19^∙^
Credit-rating	86.13 ± 3.92	85.61 ± 3.95^∙^	2414.60 ± 123.66	3226.25 ± 131.46^∙^
German-credit	74.98 ± 3.63	73.13 ± 4.00^∙^	4410.11 ± 114.94	6007.28 ± 124.30^∙^
Ecoli	83.77 ± 5.96	83.24 ± 5.98^∙^	1498.86 ± 62.27	1806.26 ± 70.98^∙^
Hayes-roth	78.75 ± 9.57	76.81 ± 9.16^∙^	275.09 ± 47.90	311.32 ± 57.05^∙^
Heart-c	81.21 ± 6.37	79.99 ± 6.65^∙^	1230.14 ± 54.80	1510.57 ± 52.56^∙^
Horse-colic	84.53 ± 5.30	83.80 ± 6.11^∙^	940.07 ± 66.64	1337.60 ± 75.73^∙^
Ionosphere	93.90 ± 4.05	94.02 ± 3.83	590.63 ± 65.62	706.79 ± 73.17^∙^
Iris	94.47 ± 5.11	94.47 ± 5.02	152.58 ± 108.04	197.80 ± 141.31^∙^
Lymphography	81.65 ± 9.45	81.46 ± 9.39	858.42 ± 46.50	1022.67 ± 39.68^∙^
Page-blocks	97.02 ± 0.74	97.07 ± 0.69	1396.63 ± 237.03	2086.89 ± 399.10^∙^
Pima	74.92 ± 3.94	74.03 ± 3.58^∙^	2391.95 ± 764.16	2910.31 ± 936.70^∙^
prnn-fglass	78.13 ± 8.06	77.99 ± 8.44	1280.14 ± 43.85	1410.84 ± 39.59^∙^
Vote	95.70 ± 2.86	95.33 ± 2.97	177.36 ± 86.10	281.62 ± 140.60^∙^
